# Micro- and nanoplastic exposure as an emerging risk factor for depressive-like phenotypes across species: a systematic review

**DOI:** 10.3389/ftox.2026.1817678

**Published:** 2026-05-28

**Authors:** Donato Morena, Damiano Marchesini, Monica La Greca, Letizia Biso, Matteo Lippi, Matteo Scopetti, Emanuela Turillazzi, Vittorio Fineschi

**Affiliations:** 1 Department of Anatomical, Forensic and Orthopedic Sciences, Sapienza University of Rome, Rome, Italy; 2 Department of Translational Research and New Technologies in Medicine and Surgery, University of Pisa, Pisa, Italy; 3 Department of Medical Surgical Sciences and Translational Medicine, Sapienza University of Rome, Rome, Italy; 4 Department of Surgical Pathology, Medical, Molecular and Critical Area, University of Pisa, Pisa, Italy

**Keywords:** depression, depressive-like behavior, gut-brain axis, microplastics, nanoplastics, MNPs, neuroinflammation, neurotoxicity

## Abstract

The pervasive environmental presence of micro- and nanoplastics (MNPs), combined with their ability to cross biological barriers and accumulate in the brain, has raised significant concerns regarding their neurotoxic potential. While neuroinflammatory and neurodegenerative effects are increasingly documented, a focused synthesis of their impact on depression-relevant behaviors is lacking. This systematic review evaluates the evidence linking MNPs exposure to depressive-like phenotypes across species. Following PRISMA guidelines, nine animal studies and one human cross-sectional study met the inclusion criteria. Rodent models exposed to MNPs, primarily polystyrene, showed consistent induction of depressive-like behaviors, whereas aquatic models exhibited neurobehavioral alterations compatible with depression-relevant phenotypes. Effects were dose-dependent and influenced by particle characteristics, exposure window, and sex. Preliminary human data suggest a positive association between estimated environmental MNPs exposure and depressive symptoms. Although evidence remains limited and methodologically heterogeneous, converging findings across experimental models indicate that MNPs exposure may represent a potential emerging risk factor for depression-related outcomes, possibly mediated by neuroinflammatory processes and neuronal damage. Future research requires standardized behavioral assessments, environmentally relevant exposure models, and longitudinal human studies with direct exposure biomonitoring to establish causality and public health relevance.

## 1 Introduction

Depressive disorders are a leading global cause of disability and arise from complex interactions among genetic vulnerability, neurobiological dysregulation, and environmental exposures.

In recent years, environmental contaminants capable of reaching the central nervous system (CNS) have received growing attention for their potential influence on mental health. Micro- and nanoplastics (MNPs) have emerged as contaminants of particular concern due to their persistence, ubiquity, and demonstrated ability to accumulate in multiple human tissues, including blood, placenta, amniotic fluid, cerebrospinal fluid, and brain (Leslie et al., 2022; Kumari et al., 2025; Guan et al., 2023; Cui et al., 2024; Shao et al., 2025).

Plastics are manufactured through polymerization processes involving diverse monomers and additives, resulting in materials characterized by durability, chemical stability, and corrosion resistance (Yang H. et al., 2022). Global production and inadequate end-of-life management continue to drive the generation and environmental fragmentation of synthetic polymers, thereby increasing the formation of secondary MNPs across multiple environmental compartments (Geyer, 2020; Pfohl et al., 2025; Pfohl et al., 2022; Xiang et al., 2023).

These particles are widely documented across terrestrial soils (350–1,604 particles/kg), agricultural soils (88–2,830 particles/kg), atmospheric deposition (2–1,008 particles/m^2^/d), sea surface waters, sea ice, and deep marine ecosystems (Wright et al., 2020; de Haan et al., 2019; Andrady, 2017; Koelmans et al., 2019; Büks and Kaupenjohann, 2020; Allen et al., 2022). Plastics are classified by size: particles larger than 5 mm are defined as macroplastics, those smaller than 5 mm are termed microplastics (MPs), and particles smaller than 1 μm are referred to as nanoplastics (NPs).

More broadly, MNPs are solid, insoluble particles that originate either as primary particles (e.g., synthetic fibers and industrial microbeads) or as secondary fragments generated through the environmental degradation of macroplastics. This degradation occurs *via* processes such as hydrolysis, ultraviolet (UV) photodegradation, elevated temperatures, mechanical weathering, and microbial activity. Recent work confirms that environmental stressors such as UV irradiation and temperature fluctuations promote photo-oxidation and fragmentation, leading to the release of secondary MNPs’ fragments across various polymer types (Pfohl et al., 2025; Pfohl et al., 2022; Xiang et al., 2023).

MPs are the most abundant and originate from either primary or secondary sources (GESAMP; Hartmann et al., 2019). Surface-water surveys consistently show that low-density polymers dominate floating MPs. In the Western Mediterranean Sea, polyethylene (PE) accounts for approximately 54.5% and polypropylene (PP) for 16.5% of floating MPs. At the same time, other polymers such as polystyrene (PS), polyvinyl chloride (PVC), polyester, and polyamides occur at lower proportions (de Haan et al., 2019). Globally, reviews indicate that PE and PP are the most frequently reported polymers in surface waters due to their high production volumes and buoyancy relative to seawater (Andrady, 2017; Koelmans et al., 2019).

Human exposure to MNPs is continuous and multidimensional, occurring predominantly through the ingestion of contaminated food and water, as well as *via* inhalation, dermal, and ocular routes (Leslie et al., 2022; Kumari et al., 2025; Guan et al., 2023; Kha et al., 2024; Kadac-Czapska et al., 2024). The extent to which inhaled or ingested MNPs are absorbed and systemically distributed in humans remains, however, incompletely understood, partly due to substantial analytical and methodological variability across studies and the limited availability of standardized exposure biomarkers (Shao et al., 2025; Cho and Choi, 2021). Nevertheless, a growing body of evidence increasingly highlights the potential risks associated with human exposure to MNPs (Marfella et al., 2024; Prüst et al., 2020; Lamoree et al., 2025; Fang et al., 2025).

Their small size and high surface-area-to-volume ratio enhance environmental mobility, bioavailability, and the potential for cellular uptake. MNPs have been shown to cross biological barriers (e.g., gut epithelium, blood–brain, placental, and blood–testis barriers) and distribute to internal organs (Bai CL. et al., 2024; Khan and Jia, 2023; Ma et al., 2025; Araújo et al., 2025; Gao et al., 2025; Alqahtan et al., 2023; Yang W. et al., 2022). Once internalized, MNPs can localize in intracellular organelles and trigger processes including oxidative stress, inflammation, apoptosis, and other toxicological pathways (Khan and Jia, 2023; Forutan et al., 2025).

Experimental studies and narrative syntheses indicate that exposure to MNPs can disrupt gastrointestinal function, reproductive and endocrine homeostasis, and immune activity, frequently through the induction of oxidative stress and inflammation (Ma et al., 2025; Araújo et al., 2025; Gao et al., 2025). For instance, exposure to NPs alters gut integrity, triggers gut inflammation, and perturbs the gut–brain axis in animal models (Sun et al., 2024).

*In vitro* studies demonstrate that NPs induce reactive oxygen species (ROS) production, mitochondrial dysfunction, NF-κB activation, necroptosis, cytokine release, and neuronal damage mediated by microglia-derived factors (Shan et al., 2022; Bai H. et al., 2024).

The blood-brain barrier (BBB) represents the primary route for MNPs to access the CNS (Yang D. et al., 2022; Kopatz et al., 2023). MNPs can cross the BBB through multiple mechanisms, including interference with tight junction proteins (Bai H. et al., 2024), utilization of cellular transport pathways such as endocytosis and macropinocytosis in endothelial cells (Ma et al., 2024), and transcytosis across the endothelial layer (Cho et al., 2024). Furthermore, neuroinflammation itself can exacerbate BBB permeability, thereby enhancing the uptake and transport of MNPs into the CNS (Cho et al., 2024).

The ability of MNPs to cross the BBB, as well as their biological effects, depends on particle size, surface chemistry, and exposure duration. Among these factors, size is a key determinant, as smaller particles consistently exhibit greater uptake and translocation across the BBB in experimental models (Cho et al., 2024). Accordingly, NPs display a higher capacity for BBB penetration than MPs (Yang ZS. et al., 2022; Waring et al., 2018; Zhao et al., 2023). In addition to size, physicochemical properties such as surface charge and chemical functionalization play a critical role in modulating BBB permeability. Positively charged NPs, in particular, tend to accumulate more readily in brain tissue, likely due to stronger interactions with negatively charged cellular membranes (Teng et al., 2022). These properties also influence biological effects. For instance, PS-NPs (∼100 nm) induce ROS production, cell cycle arrest, and apoptosis in neuronal cells, whereas larger PS-MPs (∼1 μm) exhibit minimal toxicity (Bai H. et al., 2024; Liu et al., 2022). Moreover, surface modifications, such as the introduction of cationic groups, can further enhance toxicity by promoting apoptosis- and nitric oxide (NO)–related pathways (Bai H. et al., 2024).

*In vivo*, MNPs pose acute and chronic neurotoxic threats, with species- and context-specific outcomes. Recent reviews suggest these processes may lead to neural damage, neurotransmitter imbalance, disrupted neuronal homeostasis, and, in some experimental studies, cognitive and behavioral dysfunction (Fang et al., 2025; Araújo et al., 2025; Sun et al., 2025; Vojnits et al., 2025).

Host-specific factors also strongly influence susceptibility to MNPs’ neurotoxicity (Fang et al., 2025). Developmental stages represent the most vulnerable period due to ongoing neurogenesis, synaptogenesis, and BBB maturation, with maternal exposure during pregnancy and lactation associated with persistent neurodevelopmental and behavioral alterations in offspring (Yang D. et al., 2022; So et al., 2023; Jeong et al., 2022; Tian et al., 2024; Dibbon et al., 2024; Kaur et al., 2025). In adults, age-dependent differences in behavioral and immune responses have also been reported (Gaspar et al., 2023).

Gender also influences MNPs-induced neurotoxicity: higher doses of PS-NPs (350–500 nm) cause hippocampal neuronal degeneration with greater effects in females than males, suggesting that estrogen signaling and brain sexual dimorphism may play a modulatory role (Jeong et al., 2022; Baş et al., 2023; Xian et al., 2024).

Regarding the effects of MNPs on neurodegeneration and neurotransmitter systems, animal studies have reported suppressed acetylcholinesterase (AChE) activity, along with hippocampal neuronal degeneration, dopaminergic neuron loss, and associated cognitive deficits (Fang et al., 2025; Barboza et al., 2023; Wang et al., 2022; Liang et al., 2022).

Alterations have also been observed in other neurotransmitter systems, including serotonin, glutamate, and GABA (Fang et al., 2025).

These alterations are primarily mediated by oxidative stress–related mechanisms, including increased NO, ROS, and malondialdehyde (MDA) levels, together with glutathione (GSH) depletion. In addition, disruption of the CREB/BDNF signaling pathway has been observed, leading to impaired synaptic plasticity (Lee et al., 2022; Reddy et al., 2021; Zarneshan et al., 2022).

From a behavioral perspective, a recent review by Asmaa et al. (Asmaa et al., 2025) systematically examined the effects of PS-MNPs in mice, focusing on neuronal oxidative stress, neuroinflammation, and anxiety-like behavior. The findings indicate that these particles induce neuronal oxidative stress, characterized by increased ROS production and lipid peroxidation along with reduced antioxidant defenses, including superoxide dismutase (SOD), catalase (CAT), and GSH. In parallel, elevated levels of neuroinflammatory markers, including TNF-α, IL-1β, IL-6, GFAP, and Iba1, have been reported, together with the emergence of anxiety-like behaviors. Notably, maternal exposure has also been associated with neurotoxic effects in offspring.

An additional mechanism underlying MNPs-induced mental and behavioral alterations involves their interaction with the gut microbiota. For example, exposure to PE-MNPs has been shown to induce anxiety-like behavior in adult mice, accompanied by gut microbiota dysbiosis and metabolic disturbances. These findings further support a mechanistic role of the microbiota–gut–brain axis in MNPs-related neurobehavioral toxicity (Chen et al., 2023).

Conversely, research directly addressing the association between MNPs exposure and depressive-like behaviors remains limited and fragmented. Experimental studies differ substantially in species, exposure route, dose, duration, life-stage vulnerability, and polymer chemistry, thereby limiting cross-study comparability and translational relevance to human risk (Prüst et al., 2020; Vojnits et al., 2025). Similarly, although existing reviews have broadly addressed MNPs-induced neurotoxicity, including neurodevelopmental, neurodegenerative, and neuroinflammatory outcomes, none have provided a focused, systematic evaluation of depression-relevant behavioral endpoints (Vojnits et al., 2025; Liu et al., 2024; Murthy, 2025).

These gaps are further compounded by inherent methodological constraints. In preclinical research, “depressive-like behavior” does not constitute a direct analogue of clinical depression but rather an operational construct derived from specific behavioral readouts approximating selected domains of affective dysfunction, particularly stress coping and behavioral despair/helplessness (More et al., 2025). These phenotypes are typically inferred from standardized assays designed to capture alterations in motivation, affect-related behavior, and stress responsivity (Singh et al., 2017).

In rodent studies, depressive-like behaviors are commonly assessed using the forced swim (FST) (Nishimura et al., 1988) and tail suspension (TST) (Can et al., 2012) tests, which evaluate immobility as an index of “behavioral despair” and are highly responsive to conventional antidepressant treatments (Castagné et al., 2010).

Additional measures, including reduced sucrose preference (Trullas and Skolnick, 1993), decreased locomotor activity (Varghese et al., 2016), or diminished exploration in novel environments (Seibenhener and Wooten, 2015; S et al., 2023), are also frequently interpreted as indicative of depression-relevant behavioral alterations.

In aquatic models such as zebrafish, endpoints including reduced exploration (e.g., the novel tank test, NTT), increased freezing or anhedonia-like states (e.g., reduced preference for light, food, conspecifics, and sexual interactions) are commonly used as species-specific indicators of affective and stress-related behavioral disruption, although they are not uniquely specific to depressive-like states (Hassani Nia and Wittamer, 2025; Stewart et al., 2012; Fontana et al., 2018; Nguyen et al., 2014).

Specifically, the FST (Nishimura et al., 1988) evaluates the duration of immobility when an animal is placed in an inescapable container of water, reflecting a passive coping strategy to acute stress, while the TST (Can et al., 2012) measures immobility when rodents are suspended by the tail, similarly interpreted as behavioral despair. The sucrose preference test is commonly used to assess anhedonia by quantifying reduced preference for a sweet solution over water (Trullas and Skolnick, 1993). Locomotor and exploratory behaviors are typically evaluated using paradigms such as the open field test (OFT) (Seibenhener and Wooten, 2015), where reduced center exploration or overall activity may indicate altered motivational or affective states.

Although more specific for anxiety-like behavior, the elevated plus maze (EPM), a test based on the conflict between avoidance of open, elevated arms and exploration of enclosed arms, indicates higher anxiety-like/affective impairment when open-arm time and entries are reduced (Pellow et al., 1985).

In zebrafish, the NTT assesses vertical exploration and habituation to a novel environment, with increased bottom-dwelling or freezing behavior more cautiously interpreted as an index of stress-related or anxiety-like responding, and only more indirectly as relevant to depression-related behavioral domains (Stewart et al., 2014). The light-dark box test (LDB) assesses affective states by measuring a fish’s natural avoidance of illuminated areas in a two-compartment apparatus (light vs. dark) (Campos-Cardoso et al., 2023). Reduced exploration of the light zone (less time and fewer transitions) indicates increased anxiety-/depression-like behavior, while greater light exploration suggests reduced stress.

Together, these assays capture partially overlapping but distinct behavioral domains and represent the core set of *in vivo* tests used to operationalize depressive-like phenotypes in experimental studies (for additional details on the behavioral tests, see Appendix A in Supplementary Material S1).

This framework informed the selection of eligible studies included in the present review, which systematically synthesizes the current evidence on the relationship between MNPs and depressive-like behavior or depressive symptoms.

## 2 Methods

This systematic review was conducted in accordance with the PRISMA guidelines (Page et al., 2021). A comprehensive literature search was conducted from database inception to September 2025 using PubMed, Scopus, and Web of Science (for the respective search string, see Appendix B in Supplementary Material S1), as well as through free-text searches of ClinicalTrials.gov and Google Scholar. Two independent reviewers (D.Mo. and D.Ma.) screened the records and extracted relevant study characteristics. Discrepancies arising during the screening or data extraction phases were resolved through discussion, with arbitration by senior review authors (E.T. and V.F.), based on the predefined inclusion criteria and the relevance of the data to the study objectives. In addition, emerging reviews and the reference lists of eligible articles were manually screened by two investigators (D.Mo. and D.Ma.).

The inclusion criteria were as follows: 1) human or animal studies; 2) studies in which MPs and/or NPs were used as exposure agents; 3) no restrictions on exposure method, timing of exposure onset, or duration of the experiment; 4) use of diagnostic or evaluative tools for depressive symptoms; and 5) for animal studies, use of validated tests designed to model depression-like behavior.

No *a priori* restrictions were imposed with respect to animal species.

Studies were excluded if they 1) were deemed out of scope; 2) were published in a language other than English; 3) were non-original articles (e.g., editorials, book chapters, letters to the editor, reviews, or meta-analyses); 4) involved *in vitro* experiments rather than whole-animal models; 5) exposed animals to a single dose only; or 6) employed non-specific behavioral tests to assess depression-like phenotypes in animal models.

The search strategy included all types of MNPs reported in the literature to the best of our knowledge. Additionally, the search was intentionally broad with respect to depressive symptoms, behaviors, and assessment tools, in order to allow a subsequent screening step to verify the presence or absence of validated, depression-specific tests.

To ensure greater methodological homogeneity in laboratory analyses, a time restriction was applied to exclude articles published before January 2000.

The protocol for this review has been registered with the International Prospective Register of Systematic Reviews (PROSPERO registration number CRD420261324756).

### 2.1 Analysis of reporting quality and risk of bias

The quality of the animal studies was assessed using the SYRCLE tool (Systematic Review Centre for Laboratory Animal Experimentation), which is adapted from Version 2 of the Cochrane risk-of-bias tool for randomized trials (RoB 2) to evaluate methodological quality and potential bias in animal intervention studies and has been previously used in similar reviews (Asmaa et al., 2025). SYRCLE’s tool includes 10 items covering sequence generation (Q1), baseline comparability or adjustment for confounders (Q2), allocation concealment (Q3), random housing (Q4), blinding of caregivers and/or investigators (Q5), random outcome assessment (Q6), blinding of outcome assessors (Q7), handling of incomplete outcome data (Q8), selective outcome reporting (Q9), and other potential sources of bias (Q10). For Q10, additional considerations included compliance with ethical standards, adequacy of methodological reporting, appropriateness of statistical analyses, disclosure of funding and conflicts of interest, and any other evident sources of bias.

The quality assessment of the only human study included was conducted using the Joanna Briggs Institute (JBI) Critical Appraisal Checklist for Analytical Cross-Sectional Studies (Barker et al., 2023).

## 3 Results

### 3.1 Study selection

**FIGURE 1:**
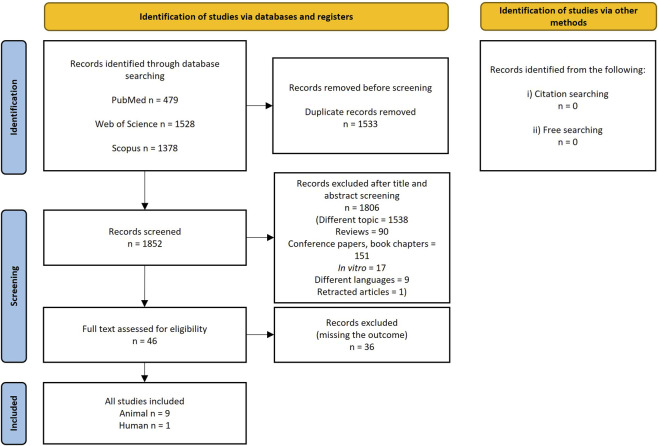
Summary of systematic review performed according to the Preferred Reporting Items for Systematic Reviews and Meta-Analysis Guidelines.

The searches identified 3,385 records. After removing duplicates, 1852 records remained for the screening of titles and abstracts. During this screening, 1,538 records were excluded because they considered different topics, 151 were conference papers, book chapters, or other non-relevant publication types, 90 were reviews, 17 were *in vitro* studies, 9 were in languages other than English, and one record was a retracted article. 46 records were selected for full-text assessment. Ultimately, 36 were missing the outcome, and 10 studies (9 animal and one human) that met the inclusion criteria were included and constituted the dataset for this study. The PRISMA flow diagram illustrating the study selection process is shown in Figure 1.

### 3.2 Bias report and quality of studies

**FIGURE 2:**
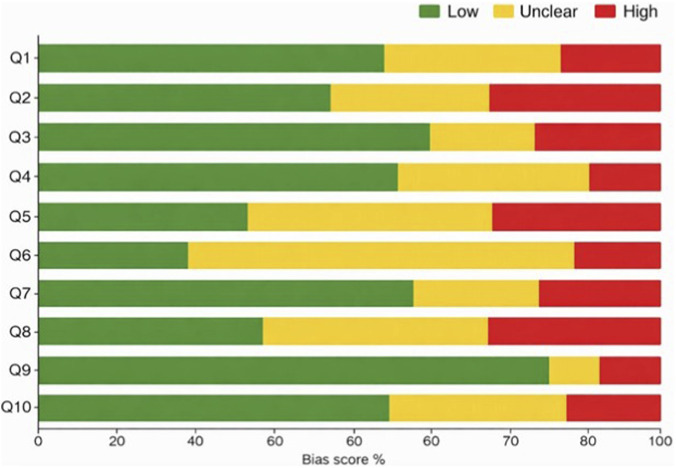
Assessment of the risk of bias of animal studies using the SYRCLE tool.

Overall, the included studies demonstrated a mixed RoB profile across SYRCLE domains (Figure 2). Most studies were judged at low risk for sequence generation, baseline comparability, allocation concealment, blinding of outcome assessors, and selective outcome reporting. In contrast, performance-related domains, and particularly blinding of caregivers and/or investigators, were frequently rated as unclear or high risk. Several domains, including random housing, random selection for outcome assessment, and handling of incomplete outcome data, were not fully or clearly explained. Collectively, the RoB profile indicates acceptable methodological quality in core design aspects but highlights recurrent limitations in blinding procedures and reporting transparency, which may affect internal validity. A comprehensive evaluation of each single study can be found in the Supplementary Material (Appendix C–Supplementary Material S1).

According to the JBI tool, the only human study retrieved, conducted by Luo and Lin (2025), demonstrated high methodological quality, meeting 7 of 8 appraisal criteria. The primary limitation concerned the indirect assessment of MPs’ exposure, which may have introduced exposure misclassification. Additionally, the possibility of residual confounding cannot be excluded. Taking these factors into account, the overall risk of bias was judged to be moderate to low.

### 3.3 Characteristics of the selected studies

The systematic review encompassed nine animal experimental studies (Ma et al., 2024; Shin et al., 2023; Liu et al., 2023; Guo et al., 2025; Wang et al., 2025; Teng et al., 2025; Yang et al., 2025; Su et al., 2025; Feng et al., 2024) and one human cross-sectional investigation (Luo and Lin, 2025), reflecting a diverse yet convergent approach to investigating the neurobehavioral and neurobiological consequences of MNPs exposure.

Among animal studies, the body of evidence was predominantly derived from rodent models, with a comparatively limited representation of aquatic species, including zebrafish and marine medaka.

Mammalian studies (n = 6) were predominantly employed, using C57BL/6, BALB/c, and ICR mice, with exposure initiated at various life stages: gestation (Shin et al., 2023; Guo et al., 2025), adolescence (Su et al., 2025), or adulthood (Ma et al., 2024; Liu et al., 2023; Wang et al., 2025).

Exposure routes were primarily oral gavage (doses: 1–50 mg/kg/day) or drinking water (5–20 mg/L), with durations ranging from 28 days (Ma et al., 2024) to 6 months (Liu et al., 2023). PS was the most studied polymer, with particles ranging from 25 nm NPs (Liu et al., 2023) to 1 μm MPs (Yang et al., 2025), and two studies specifically compared surface charge effects (PS, PS-COOH, PS-NH_2_) (Ma et al., 2024; Teng et al., 2025).

Aquatic models (zebrafish, marine medaka) enabled full life cycle and multigenerational assessments at environmentally relevant concentrations (10–250 μg/L) (Teng et al., 2025; Yang et al., 2025; Feng et al., 2024).

Neurobehavioral assessment consistently relied on depression-specific tests in rodents and species-specific behavioral paradigms in fish, which were interpreted more cautiously as affective- or stress-related endpoints relevant to, but not specific for, depressive-like phenotypes. These approaches were complemented by multi-omics and histopathological analyses.

Complementing animal evidence, one cross-sectional study evaluated estimated airborne and drinking-water microplastic exposure in 1,420 young adults and examined depressive symptoms using a validated psychiatric screener (Luo and Lin, 2025).

A summary of the characteristics of studies included in the present systematic review is presented in Table 1.

**TABLE 1:** Summary characteristics of studies included in the present systematic review.

Study references	Country	Sample size (animals)	Sex	Age (w)	Particle category	Size (μm)	Exposure route	Dose	Duration (d)	Frequency	Measured outcomes*
Su et al. (2025)	China	15–30 (mice)	M	3 PND21 (at start)	NP	Not specified (Nanoscale)	OG	10 mg/kg	60	Daily	DLB (TST, FST), ALB (OFT, EPM), neuronal morphology, synaptic transmission, astrocyte activation, glutamate transporter (EAAT2) expression
Shin et al. (2023)	Korea	79 (mice)	M-F	0–16 (PND 1–113)	NP	0.04/0.193	DW	5, 10 mg/L	104 (E9.5 to P113)	Daily	DLB (FST, TST), ALB (OFT, EPM), Social Deficit
Liu et al. (2023)	China	40 (mice)	M	5 (at start)	NP	0.025	OG	10, 25, 50 mg/kg	180	Daily	DLB (TST), ALB (OFT) transcriptomic changes in PFC
Guo et al. (2025)	China	Offspring: 20 M + 20 F per group [from exposed dams] (mice)	M-F	Adult (tested at∼8)	NP	0.08	DW	10, 20 mg/kg (to dams)	25 (GD17–PND21)	Daily (to dams)	DLB (TST), gut dysbiosis, hippocampal metabolite changes
Wang et al. (2025)	China	Not explicitly stated [multiple cohorts for behavior/omics] (C57BL/6 mice)	M	6 at start	NP	0.05	OG	25 mg/kg	30	Daily	ALB, DLB (FST, TST), NI, atherosclerosis
Ma et al. (2024)	China	32 (BALB/c mice)	M	4	NP (PS, PS-COOH, PS-NH_2_)	∼0.1	OG	1 mg/day (∼1.8 × 10^12^ particles/day)	28	Daily	ALB (OFT, EPM), DLB (FST), Social Deficit, mitochondrial dysfunction
Teng et al. (2025)	China	≥24–32 per sex per group (Zebrafish - *Danio rerio*)	M-F	Full life cycle (embryo to adult)	NP (PS, PS-NH_2_, PS-COOH)	44, 51, 50 nm	WE	10 μg/L	120	Continuous	ALB, DLB, Cognitive deficit, Social Deficit, brain damage
Yang et al. (2025)	China	60 per group (Zebrafish - *Danio rerio*)	M-F	Adult	MP	0.6–1.0 (hydrodynamic: 1,220–1,656 nm)	WE	25 μg/L, 250 μg/L	40	Continuous (renewed every 48 h)	DLB (NTT, LDB), neuroinflammation, circadian disruption, neurotransmitter imbalance
Feng et al. (2024)	China	50 (25M + 25F) per tank, 3 replicates (*Oryzias melastigma*)	M-F	Adult (4-month-old) and larvae	MP-NP	MP: ≤5 μm; NP: ≤1 µm (estimated)	WE	MP/NP: 200 μg/L; TPT: 200 ng/L	F0: 42; F1-F2: multigenerational	Continuous (water renewal)	DLB, oxidative stress in brain, energy metabolism in liver, gonadal development, multigenerational effects

Abbreviations: ALB, anxiety-like behavior; DLB, depressive-like behavior; DW, drinking water; EPM, elevated plus maze; F, female; FST, forced swim test; GD, gestational day; LDB, light/dark box; M, male; MP, microplastics; NI, neuroinflammation; NP, nanoplastics; NTT, novel tank test; OFT, open field test; OG, oral gavage; PND, postnatal day; TST, tail suspension test; w, week; d, day; WE, waterborne exposure.

### 3.4 Effects on depressive-like behavior

The available evidence indicates that MNPs exposure, especially in rodent models, can induce depression-relevant behavioral alterations, though the effect shows dose-dependence and may be influenced by exposure window and particle characteristics.

For rodent studies, the majority reported significantly increased immobility in depression-specific tests such as FST and TST, particularly at higher exposure doses or prolonged exposure durations (Ma et al., 2024; Shin et al., 2023; Guo et al., 2025; Wang et al., 2025; Su et al., 2025). Also, exposure to charged NPs (PS, PS-COOH, PS-NH_2_) at an environmentally relevant dose was considered a risk factor (1 mg/day for 28 days) (Ma et al., 2024). Some models showed mixed or dose-dependent findings, including nonsignificant changes in the TST despite other depression-related indications (Liu et al., 2023), significant immobility increase only at the highest exposure concentration (Shin et al., 2023), or sex-specific perinatal exposure effects (e.g., significant immobility in TST only in high-dose males) (Guo et al., 2025), suggesting potential complex dose-response dynamics or adaptation.

In aquatic models, neurobehavioral alterations were assessed using zebrafish-specific paradigms, in which MNPs exposure induced reduced exploration, increased freezing or bottom-dwelling, and altered stress responses; although these outcomes were classified by the authors as depressive-like endpoints, they may also reflect anxiety-related behaviors, stress reactivity, or more general toxic effects (Teng et al., 2025; Yang et al., 2025). Similarly, marine medaka exhibited reduced swimming activity and increased inactivity across the F0–F2 generations; these findings were interpreted by the investigators as behavioral depression but should be considered with caution due to their limited phenotypic specificity (Feng et al., 2024).

The human observational evidence aligned with animal findings: higher estimated MPs exposure, particularly airborne, was significantly associated with greater odds of depressive symptoms, showing a dose-response pattern (OR = 1.38, 95% CI: 1.21–1.57) (Luo and Lin, 2025).

### 3.5 Effects on neuronal state (neuroinflammation, glial activation, neuronal oxidative stress)

When analyzing neuronal state, some studies found no changes in glial populations or apoptosis markers (Shin et al., 2023). Structural neuronal alterations, including dendritic spine loss, synaptic thinning, neuronal degeneration, and mitochondrial swelling, were identified in both rodent hippocampus and zebrafish brain tissue (Ma et al., 2024; Guo et al., 2025; Teng et al., 2025). The human study did not measure oxidative stress or inflammatory biomarkers, but its findings of increased depressive symptoms are compatible with mechanistic pathways reported in animal studies (Luo and Lin, 2025).

MNPs exposure was associated with a range of neurobiological alterations, summarized in a triad of interconnected neurobiological disturbances: oxidative stress, neuroinflammation, and neuronal damage. Nevertheless, findings varied by model, dose, and particle type; regarding oxidative stress, evidence is mixed, with a reliance on indirect measures. The most direct evidence comes from *ex vivo* neuronal models showing increased mitochondrial ROS (mitoSOX) and dysfunction (Ma et al., 2024) and from fish studies reporting elevated brain MDA and NO with impaired antioxidant defenses (SOD, GSH) (Feng et al., 2024). Transcriptomic analyses in rodents and metabolic pathway enrichment further indicated activation of oxidative stress–related processes without direct biochemical quantification (Su et al., 2025; Liu et al., 2023; Wang et al., 2025).

Neuroinflammation and glial activation were observed in multiple models through upregulation of microglial activation (Iba1+, M1 polarization) and astrogliosis (increased GFAP, hypertrophic morphology) (Ma et al., 2024; Su et al., 2025), supported by upregulation of pro-inflammatory cytokines (IL-1β, IL-6, TNF-α) (Yang et al., 2025), inflammatory gene expression (Teng et al., 2025), and inflammatory pathway enrichment in omics data (Wang et al., 2025).

However, one well-controlled study found no evidence of glial activation or changes in inflammatory markers despite observing behavioral deficits, proposing an alternative mechanism *via* GABAergic synapse disruption (Shin et al., 2023). This highlights that neuroinflammation may be a common, but not obligatory, pathway in MNPs-induced neurobehavioral toxicity.

Structural and functional neuronal alterations were identified in several animals models, including: 1) reduced dendritic spine density in the hippocampus (Guo et al., 2025); 2) ultrastructural damage: swollen neurons, fragmented mitochondria, and synaptic abnormalities (Ma et al., 2024; Guo et al., 2025; Teng et al., 2025); 3) transcriptomic and metabolomic dysregulation of pathways critical for synaptic plasticity, neurotransmission (e.g., serotonergic, dopaminergic), and neurodevelopment (Liu et al., 2023; Wang et al., 2025).

Summaries of the effects on neuronal state and depressive-like behavior, and on neuroinflammation and glial responses, are presented in Tables 2, 3, respectively.

**TABLE 2:** Effects on neuronal state and depressive-like behavior.

Study	Brain region(s) studied	Key findings related to depressive-like behavior and neuronal structural and/or functional changes
Su et al. (2025)	mPFC	• DLB: ↑ immobility in TST and in FST. • ALB: ↓ entries/time in the center in OFT; ↓ time in the open arms in EPM. • Neuronal impairment: Reduced dendritic complexity, spine density, and excitatory synaptic transmission (mEPSC frequency) in mPFC pyramidal neurons. Decreased *in vivo* neuronal calcium activity.
Shin et al. (2023)	Whole brain; NAc core, Cortex, Hippocampus.	• DLB: ↑ immobility in TST and in FST. • ALB: ↓ time/entries in the center in OFT; ↓ time in the open arms in EPM (significant at 10 mg/L). • Social deficit: ↓ sociability and social novelty. • Impaired neurodevelopment: Downregulation of key brain development genes (Fgf8, Shh, Wnt2b, Wnt3, Ccnd1, Ctmb1, Creb1) in embryonic brains (E17.5). • GABAergic system alteration: Significant decrease in Gabra2 mRNA and protein levels in both embryonic and adult (P113) brains. Reduced density of Gabra2 clusters specifically in the Nucleus Accumbens core.
Liu et al. (2023)	PFC	• DLB: behavioral tests overall suggest depression-like responses in the 50 mg/kg group, but the TST alone p > 0,05; OFT (↓ central activity), NSF (↑ feeding latency), SPT (↓ sucrose preference). RNA-seq: 987 DE mRNAs, 116 DE lncRNAs, 29 DE miRNAs. Enriched pathways: axon guidance, dopaminergic synapse, neurotrophin signaling. ceRNA network: DE lncRNAs regulate Camk2a, Camk2d, HRAS *via* miRNA sponging, linking transcriptomic dysregulation to depressive phenotype.
Guo et al. (2025)	Hippocampus (CA1), mPFC	• DLB: ↑ immobility in TST (males, high dose), ↓ latency to immobility in both sexes. Social behavior: ↓ social dominance and ↓ social interaction in both sexes. • Neuronal structure: ↓ dendritic spine density in hippocampal neurons. • Ultrastructural damage: Swollen neurons, dilated ER, fragmented mitochondria, thinner postsynaptic densities, wider synaptic clefts (TEM).
Wang et al. (2025)	Hippocampus, Amygdala	• DLB: ↑ immobility in FST and in TST. • Proteomic dysregulation: ↑ SHISA7 (synaptic plasticity, GABA); ↓ CACNG8 (AMPA receptor, neurodegeneration link). • Metabolomic dysregulation: Enrichment in serotonergic synapse and retrograde endocannabinoid signaling pathways.
Ma et al. (2024)	Whole brain/Neurons	• DLB: ↑ Immobility (FST). • ALB: ↓ Center activity (OFT); ↓ Open arm time (EPM). • Social deficit: Impaired preference/novelty. • Mitochondrial dysfunction: NPs localize in mitochondria; ↓ ATP; ↑ mitoSOX; ↓ MMP; ↓ OCR. • Apoptosis: ↑ TUNEL+ neurons; ↓ Bcl-2/Bax.
Teng et al. (2025)	Whole brain	• DLB: ↓ locomotor behaviors in PS group, including changes to active time (0.45-fold), average velocity (0.57-fold), and distance traversed (0.57-fold). • Cognitive deficit: ↑ Time in wrong zone (PS-NH2 and PS-COOH impair cognitive ability in both males and females). • Social deficit: ↓ Interaction distance (male: PS-COOH). • Brain injury: Scattering of the periventricular gray matter layer of the midbrain, colliculus mesencephali damage, and inflammatory cell infiltration (PS and PS-COOH). • Ultrastructural damage: Nuclear rupture, mitochondrial swelling, cristae loss. • BBB disruption: Broken tight junctions, ↑ permeability (female: PS, PS-COOH).
Yang et al. (2025)	Whole brain (optic tectum, telencephalon)	• DLB: NTT: ↓ time in top zone, ↓ distance traveled, ↑ latency, ↑ freezing frequency/duration. LDB: ↓ entries/time in light zone. ST: ↓ inter-individual distance (increased cohesion). • Neuronal damage/histopathology: Perinuclear vacuolation, thinning of optic nerve layer, reduction of Nissl bodies. Fluorescent PS-MPs detected in brain. • Neurotransmitter alterations: ↓ Dopamine, ↓ Acetylcholine, ↓ Serotonin, ↓ Norepinephrine, ↓ Tyrosine, ↓ Tryptophan, ↓ GABA trend.
Feng et al. (2024)	Whole brain	• DLB: F0 adults:- ME and NE (MPE/NPE alone): Depressive inactivity (ME > NE). - MTE and NTE (combined): Depressive inactivity (MTE > NTE). F1 and F2 offspring:- Depressive effects persisted in MPE/NPE groups across generations. - ME and NTE showed longer-lasting behavioral inhibition. • Neuronal: ↑ NO and MDA; ↓ SOD and GSH. ↑ Apoptosis (↓ bcl2, ↑ caspase3). Histopathological changes in gray matter (↑ nuclear density in combined groups).

Abbreviations: ALB, Anxiety-Like Behavior; ATP, adenosine triphosphate; BBB, Blood-Brain Barrier; CA1, Cornu Ammonis Area 1; DE, differentially expressed; DLB, Depressive-Like Behavior; EPM, elevated plus maze; ER, endoplasmic reticulum; FST, forced swim test; GABA, Gamma-Aminobutyric Acid; GSH, glutathione; LDB, Light-Dark Box; lncRNA, Long Non-coding RNA; mEPSC, miniature excitatory postsynaptic current; miRNA, MicroRNA; MMP, mitochondrial membrane potential; mPFC, medial prefrontal cortex; ME, exposure to microplastics; MTE, exposure to a combination of microplastics and triphenyltin; NE, exposure to nanoplastics; NAc, Nucleus Accumbens; NP, nanoplastic; NSF, Novelty-Suppressed Feeding; NTE, exposure to combination of nanoplastics and triphenyltin; NTT, novel tank test; OCR, oxygen consumption rate; OFT, open field test; PFC, prefrontal cortex; PS, polystyrene; RNA-seq, RNA, sequencing; SPT, sucrose preference test; ST, shoaling test; T, exposed to Triphenyltin; TEM, transmission electron microscopy; TST, tail suspension test; TUNEL, Terminal deoxynucleotidyl transferase dUTP, Nick-End Labeling.

**TABLE 3:** Effects on neuroinflammation and glial response.

Study	Brain region(s)	Key findings on neuroinflammation/Glial activation
Su et al. (2025)	mPFC	• Astrocyte activation: Significant increase in astrocyte marker GFAP at both mRNA and protein levels. Morphological changes indicative of a reactive state (hypertrophy). Increased *in vivo* calcium activity in astrocytes. • Glutamate dysregulation: Decreased expression of astrocyte glutamate transporters EAAT1 and EAAT2. No significant change in other glutamate-glutamine cycle proteins (GLS, VGLUT1, NR2A, GS). • Rescue by EAAT2 activation: Treatment with EAAT2 activator (LDN-212320) normalized EAAT2 levels, restored synaptic function, and alleviated depressive- and anxiety-like behaviors.
Shin et al. (2023)	Whole brain; NAc core, Cortex, Hippocampus.	No overt neuroinflammation or gliosis: Comprehensive immunohistochemical analysis showed no significant change in the number or distribution of: • Microglia (Iba1+ cells) • Astrocytes (GFAP+ cells) • Oligodendrocytes (Olig2+ cells) • No change in proliferation marker Ki67+ cells or apoptosis marker cleaved caspase-3+ cells in the brain. Mechanistic Focus: The study’s primary proposed mechanism is neurodevelopmental and synaptic, *via* disruption of GABAergic signaling (Gabra2 downregulation), rather than *via* a neuroinflammatory pathway.
Liu et al. (2023)	PFC	Not directly assessed. Focus on transcriptomic changes and behavioral outcomes. No data reported on classical neuroinflammatory markers (cytokines, GFAP, Iba1) or microglial/astrocyte activation
Guo et al. (2025)	Hippocampus (CA1), mPFC	Not directly assessed. Focus on behavioral outcomes, neuronal ultrastructure, gut microbiota, and hippocampal metabolomics. No data reported on cytokines, microglial markers (Iba1), or astrocyte activation (GFAP).
Wang et al. (2025)	Hippocampus, Amygdala	• Pro-inflammatory proteome: Enrichment in humoral immune response and apoptotic signaling. • Pro-inflammatory metabolome: Upregulated arachidonic acid metabolism (16R-HETE, PGH2) and Th17 cell differentiation pathways. • Joint pathway analysis: Enrichment in neutrophil extracellular trap formation (autoimmunity) and serotonergic synapse. • Context: NPs associated with microglial activation and pro-inflammatory state.
Ma et al. (2024)	Whole brain/Neurons	• Astrocyte activation: ↑ GFAP, hypertrophy. • Microglial activation: ↑ Iba1+, amoeboid morphology. • M1 Polarization (BV-2): ↑ CD68, ↓ CD206. • Pro-inflammatory markers: ↑ Il1b, inos, Tnf-a mRNA.
Teng et al. (2025)	Whole brain	• Gene dysregulation (male): PS-NH_2_ ↓ GFAP, TNFa; ↑ Mafbb, IL-4. PS-COOH ↑, IL-4, IL-13. • Gene dysregulation (female): PS and PS-NH_2_ ↑ Mafbb, IL-4, IL-13. PS-NH_2_ ↑ GFAP. PS-COOH ↑ IFN. • Histopathology: Inflammatory cell infiltration. • Microglial dysregulation: Altered Mafbb expression.
Yang et al. (2025)	Whole brain (optic tectum, telencephalon)	• Glial markers/inflammation: ↑ IL-6 (4.0-fold), ↑ IL-1β (1.9-fold), TNF-α unchanged. Cortisol trend ↑ (non-significant). Microglial/astrocyte activation: *In vitro* (HMC3 microglia): ↑ CD68, ↑ CD16 (M1 polarization), ↑ IL-6, ↑ IL-1β. Morphological changes (vesicle accumulation). • Circadian rhythm disruption: ↑ per1b, per2, per3, cry1a, cry2 in brain; ↑ CRY2, PER3 in microglia.
Feng et al. (2024)	Whole brain	• Oxidative and apoptotic dysregulation: NP > MP in oxidative damage; NT > MT in combined oxidative/apoptotic effects. • Energy metabolism disruption: Glycolysis and TCA cycle inhibited (NT > MT); ATP ↓ in T, MT, and NT groups. • Multigenerational persistence: Oxidative and metabolic disturbances observed in F1 and F2.

Abbreviations: ATP, adenosine triphosphate; BV-2, Microglial Cell Line BV-2; CD16, Cluster of Differentiation 16; CD206, Cluster of Differentiation 206; CD68, Cluster of Differentiation 68; CRY2, Cryptochrome 2; EAAT1, Excitatory Amino Acid Transporter 1; EAAT2, Excitatory Amino Acid Transporter 2; F, female; GFAP, glial fibrillary acidic protein; HMC3, Human Microglial Cell Line 3; IFN, interferon; Il1b, Interleukin-1β; inos, Inducible Nitric Oxide Synthase; Iba1, Ionized Calcium-Binding Adapter Molecule 1; Ki67, Marker of Proliferation; mPFC, medial prefrontal cortex; M, male; M1, Pro-inflammatory Macrophage/Microglia Phenotype; MT, exposure to a combination of microplastics and triphenyltin; NF-κB, Nuclear Factor kappa-light-chain-enhancer of activated B cells; NP, nanoplastic; NT, exposure to combination of nanoplastics and triphenyltin; PFC, prefrontal cortex; PGH2, Prostaglandin H2; PS, polystyrene; PS-COOH, carboxylated polystyrene; PS-NH_2_, aminated polystyrene; ROS, reactive oxygen species; TCA, tricarboxylic acid cycle; TNF-α, tumor necrosis factor alpha; Tnf-a, Tumor Necrosis Factor Alpha (gene); TNF-α, Tumor Necrosis Factor Alpha (protein); T, exposed to Triphenyltin.

**FIGURE 3:**
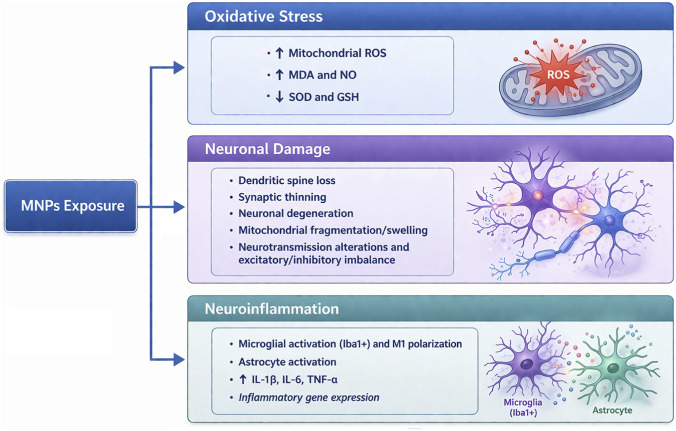
A schematic summary of all effects associated with MNPs exposure.

A schematic summary of all effects associated with MNPs exposure is presented in Figure 3.

## 4 Discussion

The collective evidence reviewed in this study indicates that MNPs exposure is associated with depression-relevant behavioral alterations across multiple animal species, with more consistent phenotypic specificity observed in rodent models compared to aquatic organisms, while preliminary human data suggest a possible association between estimated environmental exposure and depressive symptoms. These findings are in line with mechanistic disturbances reported in the toxicological literature, particularly oxidative stress, neuroinflammation, and neuronal structural damage (Prüst et al., 2020).

In experimental studies, depressive-like behavior was primarily assessed in rodents using validated paradigms, including the FST and tail suspension test TST, with increased immobility time reported in most exposed groups. These effects were more pronounced under higher doses, prolonged exposure, or during developmental windows. In aquatic models, depression-relevant behaviors were evaluated using species-appropriate endpoints, such as reduced exploration, increased inactivity, and altered swimming patterns, interpreted cautiously as indicative of affective-like states.

Particle size ranged from nanoscale to micrometer-scale formulations, although it was rarely evaluated independently of other variables. Overall, NPs appear to exert stronger interactions with CNS compartments, likely due to enhanced systemic absorption and cellular uptake, whereas larger particles may act indirectly through peripheral inflammation or gut–brain axis modulation (Fang et al., 2025; Su et al., 2025).

In aquatic models, exposure to 50 nm PS-NPs has been associated with altered exploratory behavior, brain tissue damage, and activation of oxidative stress and inflammatory pathways, indicating combined structural and neuroinflammatory effects (Sun et al., 2024). Consistently, zebrafish larvae exposed to NPs show brain accumulation, altered electrophysiological activity, hyperactivity, and disrupted dopamine metabolism (Hwang et al., 2022), while larger fish species exhibit structural brain alterations (Mattsson et al., 2015). Across aquatic organisms, behavioral disturbances have been linked to dysregulation of multiple neurotransmitter systems, including cholinergic, dopaminergic, serotonergic, and GABAergic pathways (Avio et al., 2015; Chen et al., 2017; Ding et al., 2018; Sarasamma et al., 2020).

Across studies, MNPs exposure induces neural alterations resembling those observed in neuropsychiatric and neurodegenerative conditions, including structural damage to neurons, synapses, and the BBB, supporting a potential role in neurodegenerative processes (Wilson et al., 2023). BBB disruption represents a key intermediate event, as PS particles can alter endothelial barrier integrity in a size-dependent manner, leading under chronic exposure to structural and functional impairment accompanied by oxidative stress and neuronal damage, thereby facilitating downstream neuroinflammatory and behavioral effects (Forutan et al., 2025; Cho et al., 2024).

Neuroinflammation and oxidative stress are common pathological mechanisms shared across neurodegenerative and neurodevelopmental disorders (Ransohoff, 2016), both of which may be triggered by MNPs exposure. In animal models, autism spectrum disorder (ASD)-like phenotypes have been reported following exposure to PE-MPs, accompanied by gut microbiota alterations and increased expression of EGR-1 and ARC, genes involved in synaptic plasticity and behavioral regulation (Zaheer et al., 2022). Maternal exposure to PS has also been associated with selective particle accumulation in the offspring thalamus, suggesting interference with embryonic brain development (Yang D. et al., 2022).

Within the included studies, depressive-like behavior frequently co-occurred with structural and functional alterations in brain regions involved in mood regulation, including the medial prefrontal cortex, hippocampus, and nucleus accumbens. These changes included reduced dendritic complexity, decreased spine density, synaptic dysfunction, and altered neuronal activity, supporting a close link between behavioral outcomes and underlying neural pathology.

Depressive-like behavior was also frequently accompanied by neuroinflammatory and glial responses, including microglial and astrocytic activation, inflammatory pathway enrichment, and increased pro-inflammatory mediators. However, at least one developmental exposure study reported depressive-like phenotypes in the absence of classical neuroinflammatory markers, suggesting that neuroinflammation is a frequent but not obligatory mechanism (Shin et al., 2023).

MNPs exposure also increases pro-inflammatory cytokines, disrupts synaptic signaling, and alters region-specific inflammatory profiles, contributing to excitatory–inhibitory imbalance (Ma et al., 2024; Bhattacharyya et al., 2025; Baroni et al., 2025). These immune changes often co-occur with oxidative stress, mitochondrial dysfunction, and neurotransmitter alterations, all of which are implicated in depressive pathophysiology (Correia et al., 2023).

An additional pathogenic mechanism is the “Trojan horse” effect (Zhang et al., 2021), whereby MNPs act as carriers for environmental contaminants such as heavy metals, persistent organic pollutants, plastic additives, and bacterial endotoxins (e.g., lipopolysaccharide, LPS), thereby modifying their transport, bioavailability, and toxicity (Kang et al., 2024; Zheng et al., 2024). Experimental and review studies have shown that MNPs can adsorb and carry co-contaminants and influence their uptake and biological effects, with interaction processes depending on both particle characteristics and environmental conditions (Oger et al., 2025; Trevisan et al., 2020; Llorca and Farré, 2021). Although this mechanism may contribute to observed neurotoxicity (Fang et al., 2025), it is not consistently evaluated in experimental studies, and its quantitative relevance remains uncertain, particularly in models using pristine particles.

Emerging evidence also indicates that MNPs may exert effects *via* epigenetic mechanisms, including alterations in DNA methylation, DNA methyltransferase expression, histone regulation, and non-coding RNAs, leading to persistent changes in gene expression (Santos et al., 2022). These modifications have been associated with disrupted neurogenesis, synaptic dysfunction, and long-term transcriptional reprogramming, with potential transgenerational persistence (Wade et al., 2025), suggesting a mechanism linking environmental exposure to durable neurobehavioral outcomes.

Although extending beyond classical depression models, these findings are relevant given that several depressive-like phenotypes emerged following gestational or perinatal exposure and persisted into adulthood, sometimes with sex-dependent effects, indicating long-term vulnerability to mood-related disorders.

Developmental exposure represents a key vulnerability window. Exposure to mixed 40–193 nm PS particles has been associated with brain accumulation, altered neurodevelopmental gene expression, and persistent disruption of GABAergic signaling (e.g., Gabra2), together with anxiety- and depression-like phenotypes in offspring (Shin et al., 2023). Neonatal exposure has further been linked to impaired microglial synaptic pruning and long-term social and behavioral deficits, supporting disruption of neurodevelopmental programming during critical periods (Zou et al., 2024).

MNPs may also contribute to neuropsychiatric outcomes *via* the gut–brain–microbiota axis, a bidirectional system involving immune, endocrine, and metabolic signalling (Ghosh and Gorain, 2025). Experimental studies show intestinal oxidative stress, barrier disruption, and microbiota dysbiosis following MNPs exposure, including shifts toward pro-inflammatory bacterial profiles (Li et al., 2023; Chen et al., 2022). In rodents, maternal exposure has been associated with offspring brain dysfunction mediated by microbial imbalance, increased intestinal permeability, elevated circulating LPS, and subsequent neuroinflammation (Lee et al., 2024; Morena et al., 2025). Dysbiosis may further impair BBB integrity and facilitate translocation of microbial metabolites such as short-chain fatty acids and LPS, mechanisms linked to depressive risk (Ghosh and Gorain, 2025; Li et al., 2024).

Despite rapidly growing experimental evidence, human studies remain limited, and robust epidemiological data linking MNPs exposure to depressive disorders are still scarce (Bhattacharyya et al., 2025). Nevertheless, observational studies suggest associations with cognitive impairment (Zhu et al., 2024) and emotional and behavioral dysregulation (Dong et al., 2025). The only human cross-sectional study included here reported a significant association between higher estimated MP exposure and increased likelihood of depressive symptoms, with an exposure–response trend, providing preliminary epidemiological support for experimental findings and highlighting airborne exposure as a relevant route (Luo and Lin, 2025).

These findings raise important questions regarding the potential role of environmental MNPs contamination in the increasing prevalence of psychiatric disorders, including anxiety and depression, particularly among younger populations (Bie et al., 2024).

### 4.1 Limits

The interpretation of current evidence linking MNPs exposure to depression-related outcomes is limited by a pronounced translational gap and substantial methodological heterogeneity. Human evidence remains scarce and restricted to cross-sectional designs, precluding causal inference and leaving potential residual confounding, reverse causation, and exposure misclassification unresolved. In contrast, animal studies provide a more consistent yet model-dependent signal, associating MNPs exposure with depression-relevant behavioral alterations accompanied by neurobiological changes, including oxidative stress, neuroinflammation, synaptic dysfunction, and neuronal structural damage.

A key limitation of this study is the marked heterogeneity of experimental exposure models, which vary widely in particle size, polymer type, surface chemistry, exposure route, dose, duration, developmental timing, and species, parameters that are themselves biologically relevant determinants of toxicity. Among these, particle size appears particularly important in shaping toxicokinetics and downstream neurobiological effects. However, many studies rely on simplified and non-environmentally relevant conditions, including the use of pristine PS particles and high, acute exposure doses, which do not accurately reflect real-world human exposure scenarios. In contrast, environmental MNPs occur as complex, heterogeneous mixtures of polymers with diverse sizes, shapes, and chemical compositions, often interacting with co-contaminants that may exert “Trojan horse” effects. These discrepancies limit comparability across studies, complicate dose–response interpretation, and reduce the translational relevance of current findings for human health risk assessment. Furthermore, behavioral endpoints, particularly in aquatic models, may capture generalized stress responses rather than depression-specific phenotypes, introducing additional uncertainty in the interpretation of neurobehavioral outcomes.

## 5 Conclusion

Despite several constraints, the available evidence suggests that MNPs can access the brain, alter key neural pathways, and induce depression-relevant behavioral phenotypes in experimental models. However, evidence directly linking MNPs exposure to depressive symptoms in humans remains limited and preliminary, as it is currently based on a very small number of observational studies, primarily cross-sectional in design, which do not allow for assessment of temporality or causal inference. Strengthening this research area will require standardized behavioral paradigms, targeted mechanistic perturbation studies, environmentally relevant exposure models, and longitudinal human investigations incorporating exposure biomarkers and neuroimmune or neurochemical endpoints. Integrating these approaches will be essential for establishing causality and advancing understanding of MNPs as potential contributors to depression and other neuropsychiatric outcomes. Important insights are also expected from post-mortem studies of individuals with neurological or psychiatric disorders, aimed at investigating the prevalence and composition of MNPs within central nervous system tissues (Padovano et al., 2025; Morena et al., 2024).
