# Supplementary material for: Micro- and nanoplastic exposure as an emerging risk factor for depressive-like phenotypes across species: a systematic review
Source: Front Toxicol. 2026 May 28;8:1817678. doi: 10.3389/ftox.2026.1817678 (PMC13252949; doi:10.3389/ftox.2026.1817678)
Supplement: Supplementary file 1 [file Presentation1.pdf]

# Systematic Review: Micro- and Nanoplastic Exposure as an Emerging Risk Factor for Depressive-like Phenotypes Across Species

## *Supplementary Material*

## 1 Appendix A – Summary of tests used for depression-like behavior

**Elevated Plus Maze (EPM):**

The Elevated Plus Maze (EPM) is used to assess anxiety- and depression-like behavior in mice based on their innate conflict between exploration and aversion to open, elevated spaces. Individual mice are placed at the center of a plus-shaped apparatus elevated above the floor and consisting of two open arms and two closed arms. The time spent in open and closed arms, the number of entries into each arm, and the total distance traveled are recorded. Reduced time spent in and fewer entries into the open arms are interpreted as increased anxiety- and depression-like behavior, whereas increased open-arm exploration reflects reduced affective impairment.

**Forced Swim Test (FST)**

The Forced Swim Test is used to assess depression-like behavior based on behavioral despair. Animals are placed individually in a cylinder filled with water from which escape is not possible, and their behavior is recorded for a defined period. Immobility time, defined as the absence of active escape-directed behaviors and limited to minimal movements necessary to keep the head above water, is quantified. Increased immobility time is interpreted as a depression-like phenotype, while reduced immobility is considered indicative of antidepressant-like effects.

**Light/Dark Box (LDB)**

The Light/Dark Box (LDB) test is used to evaluate depression- and anxiety-like behavior in fish based on their natural preference for dark environments when exposed to a conflict situation. Individual fish are placed in an apparatus consisting of two compartments, one brightly illuminated and one dark, connected by an opening that allows free movement between compartments. The time spent in each compartment, latency to first enter the light compartment, and number of transitions between compartments are recorded. Reduced time spent in the light compartment and fewer transitions are interpreted as increased depression- and anxiety-like behavior, whereas increased exploration of the light compartment indicates reduced affective impairment.

**Novel Tank Test (NTT)**

The Novel Tank Test (NTT) is used to assess depression- and anxiety-like behavior in fish based on their exploratory response to a novel environment. Individual fish are placed in a vertically oriented tank filled with system water, and their behavior is recorded for a defined observation period. The tank is virtually divided into upper and lower zones. Time spent in the upper zone, latency to first entry into the upper zone, frequency of zone transitions, and total distance traveled are quantified. Increased bottom-dwelling behavior and prolonged latency to explore the upper zone are interpreted as indicators of depression- and anxiety-like behavior, whereas increased exploration of the upper zone reflects reduced affective impairment.

**Open Field Test (OFT)**

The Open Field Test is performed to assess locomotor activity and exploratory behavior, serving as a control to exclude motor impairments that could confound the interpretation of other behavioral tests. Total distance traveled and time spent in the center versus periphery is recorded. This test allows differentiation between depression-like behavior and alterations in general activity.

**Tail Suspension Test (TST)**

The Tail Suspension Test is employed as a measure of behavioral despair. Animals are suspended by the tail, and the duration of immobility is recorded during the test session. Immobility is defined as the absence of initiated movements, excluding passive swinging. An increase in immobility time is interpreted as enhanced depression-like behavior, whereas a reduction suggests antidepressant-like activity.

## 2 Appendix B – Search Strings

**PUBMED**

Search:

("microplastic*"[Title/Abstract] OR

"nanoplastic*"[Title/Abstract] OR

"plastic particle*"[Title/Abstract] OR

"plastic debris"[Title/Abstract] OR

"plastic pollution"[Title/Abstract] OR

"synthetic polymer*"[Title/Abstract] OR

"plastic nanoparticle*"[Title/Abstract] OR

"plastic microparticle*"[Title/Abstract] OR

"polystyrene"[Title/Abstract] OR

"polyethylene"[Title/Abstract] OR

"polypropylene"[Title/Abstract] OR

"polyvinyl chloride"[Title/Abstract] OR

"polyethylene terephthalate"[Title/Abstract] OR

"PS-MP"[Title/Abstract] OR

"PS-NP"[Title/Abstract] OR

"PE-MP"[Title/Abstract] OR

"PET-MP"[Title/Abstract] OR

"PP-MP"[Title/Abstract]

)

AND

(

"depress*"[Title/Abstract] OR

"depression"[Title/Abstract] OR

"depressive"[Title/Abstract] OR

"depressive-like"[Title/Abstract] OR

"mood disorder*"[Title/Abstract] OR

"anhedon*"[Title/Abstract] OR

"anhedonia"[Title/Abstract] OR

"apath*"[Title/Abstract] OR

"apathy*"[Title/Abstract] OR

"abul*"[Title/Abstract] OR

"abulia*"[Title/Abstract] OR

"forced swim"[Title/Abstract] OR

"sucrose preference”[Title/Abstract] OR

"sucrose consumption”[Title/Abstract] OR

"novelty suppressed feeding"[Title/Abstract] OR

"novelty-suppressed feeding"[Title/Abstract] OR

"novel tank "[Title/Abstract] OR

"novel object recognition"[Title/Abstract] OR

"morris water maze"[Title/Abstract] OR

"nest building"[Title/Abstract] OR

"open field "[Title/Abstract] OR

"tail suspension "[Title/Abstract] OR

"social dominance"[Title/Abstract] OR

"social interaction"[Title/Abstract] OR

"scototaxis"[Title/Abstract] OR

“progressive ratio task"[Title/Abstract]

)

**SCOPUS**

Search:

( TITLE-ABS ( microplastic* ) OR TITLE-ABS ( nanoplastic* ) OR TITLE-ABS ( "plastic particle*" ) OR TITLE-ABS ( "plastic debris" ) OR TITLE-ABS ( "plastic pollution" ) OR TITLE-ABS ( "synthetic polymer*" ) OR TITLE-ABS ( "plastic nanoparticle*" ) OR TITLE-ABS ( "plastic microparticle*" ) OR TITLE-ABS ( polystyrene ) OR TITLE-ABS ( polyethylene ) OR TITLE-ABS ( polypropylene ) OR TITLE-ABS ( "polyvinyl chloride" ) OR TITLE-ABS ( "polyethylene terephthalate" ) OR TITLE-ABS ( "PS-MP" ) OR TITLE-ABS ( "PS-NP" ) OR TITLE-ABS ( "PE-MP" ) OR TITLE-ABS ( "PET-MP" ) OR TITLE-ABS ( "PP-MP" ) ) AND ( TITLE-ABS ( depress* ) OR TITLE-ABS ( depression ) OR TITLE-ABS ( depressive ) OR TITLE-ABS ( "depressive-like" ) OR TITLE-ABS ( "mood disorder*" ) OR TITLE-ABS ( anhedon* ) OR TITLE-ABS ( anhedonia ) OR TITLE-ABS ( apath* ) OR TITLE-ABS ( apathy* ) OR TITLE-ABS ( abul* ) OR TITLE-ABS ( abulia* ) OR TITLE-ABS ( "forced swim" ) OR TITLE-ABS ( "sucrose preference" ) OR TITLE-ABS ( "sucrose consumption" ) OR TITLE-ABS ( "novelty suppressed feeding" ) OR TITLE-ABS ( "novelty-suppressed feeding" ) OR TITLE-ABS ( "novel tank" ) OR TITLE-ABS ( "novel object recognition" ) OR TITLE-ABS ( "morris water maze" ) OR TITLE-ABS ( "nest building" ) OR TITLE-ABS ( "open field" ) OR TITLE-ABS ( "tail suspension" ) OR TITLE-ABS ( "social dominance" ) OR TITLE-ABS ( "social interaction" ) OR TITLE-ABS ( scototaxis ) OR TITLE-ABS ( "progressive ratio task" ) )

**WOS**

Search:

( (TI=(microplastic* OR nanoplastic* OR plastic particle* OR "plastic debris" OR "plastic pollution" OR synthetic polymer* OR plastic nanoparticle* OR plastic microparticle* OR polystyrene OR polyethylene OR polypropylene OR "polyvinyl chloride" OR "polyethylene terephthalate" OR "PS-MP" OR "PS-NP" OR "PE-MP" OR "PET-MP" OR "PP-MP"))

OR

(AB=(microplastic* OR nanoplastic* OR plastic particle* OR "plastic debris" OR "plastic pollution" OR synthetic polymer* OR plastic nanoparticle* OR plastic microparticle* OR polystyrene OR polyethylene OR polypropylene OR "polyvinyl chloride" OR "polyethylene terephthalate" OR "PS-MP" OR "PS-NP" OR "PE-MP" OR "PET-MP" OR "PP-MP"))

)

AND

(

(TI=(depress* OR depression OR depressive OR "depressive-like" OR "mood disorder*" OR anhedon* OR anhedonia OR apath* OR apathy* OR abul* OR abulia* OR "forced swim" OR "sucrose preference" OR "sucrose consumption" OR "novelty suppressed feeding" OR "novelty-suppressed feeding" OR "novel tank" OR "novel object recognition" OR "morris water maze" OR "nest building" OR "open field" OR "tail suspension" OR "social dominance" OR "social interaction" OR scototaxis OR "progressive ratio task"))

OR

(AB=(depress* OR depression OR depressive OR "depressive-like" OR "mood disorder*" OR anhedon* OR anhedonia OR apath* OR apathy* OR abul* OR abulia* OR "forced swim" OR "sucrose preference" OR "sucrose consumption" OR "novelty suppressed feeding" OR "novelty-suppressed feeding" OR "novel tank" OR "novel object recognition" OR "morris water maze" OR "nest building" OR "open field" OR "tail suspension" OR "social dominance" OR "social interaction" OR scototaxis OR "progressive ratio task")))

## 3 Appendix C – Evaluation of the risk of bias (RoB) of each individual study using the SYRCLE risk-of-bias tool

Q, Question; Q1, Was the allocation sequence adequately generated and applied ?; Q2, Were the groups similar at baseline or were they adjusted for confounders in the analysis ?; Q3, Was the allocation adequately concealed ?; Q4, Were the animals randomly housed during the experiment ?; Q5, Were the caregivers and/or investigators blinded from knowledge of which intervention each animal received during the experiment ?; Q6, Were animals selected at random for outcome assessment ?; Q7, Was the outcome assessor blinded ?; Q8, Were incomplete outcome data adequately addressed ?; Q9, Are reports of the study free of selective outcome reporting ?; Q10, Was the study free of other problems that could result in a high risk of bias? (for Q10 make considerations aside from other eventually noted methodological limitations; in other words, consider if the study followed ethical guidelines, provided detailed methods, used appropriate statistical analyses, disclosed funding, and reported no conflicts of interest, as well as any other obvious sources of bias

**Su et al. 2025** (1)

| Q | Binary response | Justification |
|---|---|---|
| **Q1** Allocation sequence adequately generated/applied? | **No** | The article does not describe how animals were assigned to groups (e.g., random sequence generation method). It only states that mice were divided into control and NPS groups without details on allocation. |
| **Q2** Groups similar at baseline / confounder control? | **Yes** | The study reports no significant difference in body weight between groups at baseline (Fig. 1B), and behavioral tests confirmed no difference in locomotor activity, suggesting groups were similar at baseline. |
| **Q3** Allocation adequately concealed? | **No** | There is no mention of allocation concealment procedures (e.g., sealed envelopes, centralized randomization) in the methods section. |
| **Q4** Animals randomly housed? | **No** | The article does not specify whether housing was randomized or if litter/cage effects were controlled for. |
| **Q5** Caregivers/investigators blinded? | **Yes** | Authors state that behavioral experiments were conducted under *double-blind* conditions. |
| **Q6** Animals randomly selected for outcome assessment? | **No** | The article does not explicitly state that animals were randomly selected for outcome assessments such as behavioral tests, histology, or electrophysiology. |
| **Q7** Outcome assessors blinded? | **Yes** | The use of "double-blind" procedures implies that outcome assessors were blinded to group assignments during data collection and analysis. |
| **Q8** Incomplete outcome data adequately addressed? | **Unclear** | The article does not mention missing data, dropouts, or how incomplete outcomes were handled. Sample sizes are provided but without attrition explanation. |
| **Q9** Free from selective outcome reporting? | **Yes** | The study appears to report all planned outcomes (behavioral, molecular, electrophysiological) as described in the methods, with no obvious omission of pre-specified results. |

| Q | Binary response | Justification |
|---|---|---|
| **Q10** Free from other high-risk bias? | **Yes** | Aside from the methodological limitations noted in previous questions, the study adhered to ethical guidelines, obtained institutional oversight, and disclosed no apparent conflicts of interest, funding biases, or ethical violations. |

**Shin et al. 2023** (2)

| Q | Binary response | Justification |
|---|---|---|
| **Q1** Allocation sequence adequately generated/applied? | **Yes** | The article states: "Pregnant mice were randomly divided into three groups" (Section 2.2) and "Each experiment was performed in a blinded and randomized manner. Animals were randomly assigned to different experimental groups" (Section 2.8). This indicates random allocation. |
| **Q2** Groups similar at baseline / confounder control? | **Yes** | The study reports no significant difference in body weight between groups at baseline (Fig. 1B), and behavioral tests confirmed no difference in locomotor activity, suggesting groups were similar at baseline. |
| **Q3** Allocation adequately concealed? | **No** | There is no mention of allocation concealment procedures (e.g., sealed envelopes, centralized randomization) in the methods section. |
| **Q4** Animals randomly housed? | **No** | The article does not specify whether housing was randomized or if litter/cage effects were controlled for. |
| **Q5** Caregivers/investigators blinded? | **Yes** | The authors state: "testing was conducted by laboratory technicians blinded to the mouse group information" (Section 2.7) and "Each experiment was performed in a blinded and randomized manner" (Section 2.8). |
| **Q6** Animals randomly selected for outcome assessment? | **Yes** | The article states: "Mouse offspring were randomly selected for behavioral testing" (Section 2.7). |
| **Q7** Outcome assessors blinded? | **Yes** | As above, researchers were blinded during behavioral testing (Section 2.7), and blinded analysis is implied for other endpoints. |
| **Q8** Incomplete outcome data adequately addressed? | **Unclear** | Sample sizes are given for each experiment, but there is no mention of attrition, dropouts, or how missing data were handled. |
| **Q9** Free from selective outcome reporting? | **Yes** | All planned behavioral, molecular, and histological outcomes described in the methods appear to be reported in the results, including negative findings (e.g., no effect on cognitive function). |
| **Q10** Free from other high-risk bias? | **Yes** | Aside from the noted limitations (allocation concealment, housing randomization, incomplete data handling), the study followed ethical guidelines, reported standardized behavioral protocols, used appropriate statistics, and disclosed funding sources without obvious conflicts. |

**Liu et al. 2023** (3)

| Q | Binary response | Justification |
|---|---|---|
| **Q1** Allocation sequence adequately generated/applied? | **No** | The authors state: "A total of 40 mice were divided into a control group, a low-dose group (10 mg/kg bw PS NPs), a middle-dose group (25 mg/kg bw PS NPs), and a high-dose group (50 mg/kg bw PS NPs)". No method of randomization is described. |
| **Q2** Groups similar at baseline / confounder control? | **Yes** | All mice were male C57BL/6, 5 weeks old at the start, and housed under identical conditions. Baseline characteristics (age, sex, strain) were uniform, and no differences were reported. Statistical analyses (one-way ANOVA) were used to compare groups post-exposure. |
| **Q3** Allocation adequately concealed? | **No** | No description of allocation concealment is provided. The grouping is mentioned without details on how assignment was concealed from researchers. |
| **Q4** Animals randomly housed? | **No** | Housing randomization is not described. Mice were housed in individually ventilated cages, but no information is given on random cage assignment or litter distribution. |
| **Q5** Caregivers/investigators blinded? | **Unclear** | Blinding is not mentioned for the administration of treatments or daily care. The article does not state whether caregivers were aware of group assignments. |
| **Q6** Animals randomly selected for outcome assessment? | **Unclear** | Outcome assessment procedures (behavioural tests, tissue collection, sequencing) are described, but the authors do not state that animals or samples were selected at random for measurements or sequencing subsets. |
| **Q7** Outcome assessors blinded? | **Unclear** | Blinding of outcome assessors is not mentioned for behavioral tests or molecular analyses. |
| **Q8** Incomplete outcome data adequately addressed? | **Unclear** | The manuscript does not report any attrition, dropouts, deaths, or how missing data (if any) were handled, nor does it provide an intention-to-treat or similar approach, only that 40 mice were divided into groups and the tests performed. Absence of reporting means incomplete data handling was not addressed |
| **Q9** Free from selective outcome reporting? | **Yes** | All planned outcomes (behavioral tests, RNA sequencing, qRT-PCR validation) appear to be reported. Supplementary tables are referenced, and negative/positive findings are presented. |
| **Q10** Free from other high-risk bias? | **No** | Ethical approval, funding, conflict-of-interest statements, and standard statistical methods are provided (ethics approval by Hebei Medical University; funding and “no conflict of interest” are declared; statistical tests described). However, the authors did not report a sample-size/power calculation, did not preregister a protocol, and omitted key design safeguards (randomisation and blinding), which are additional sources of potential bias beyond the items explicitly asked about |

**Guo et al. 2025** (4)

| Q | Binary response | Justification |
|---|---|---|
| **Q1** Allocation sequence adequately generated/applied? | **Unclear** | While the authors state that pregnant females were "randomized into three experimental groups", the method of randomization is not described, making it unclear if the sequence was adequately generated. |
| **Q2** Groups similar at baseline / confounder control? | **Yes** | The study used offspring from randomly assigned dams and conducted tests on age-matched adults, with statistical adjustments (ANOVA, LSD test) applied, suggesting baseline comparability. |
| **Q3** Allocation adequately concealed? | **No** | The article does not mention any method of allocation concealment, such as sealed envelopes or central randomization, so concealment cannot be confirmed. |
| **Q4** Animals randomly housed? | **Yes** | Mice were adaptively housed under controlled conditions, and offspring were "randomly selected" for behavioral assessments, implying random housing. |
| **Q5** Caregivers/investigators blinded? | **Yes** | The authors explicitly state that "the researchers were blinded to the groups" during behavioral testing. |
| **Q6** Animals randomly selected for outcome assessment? | **Yes** | The authors specify that "10 male and 10 female offspring from each group were randomly selected for behavioral assessments". |
| **Q7** Outcome assessors blinded? | **Yes** | Blinding is confirmed for behavioral tests and is implied for other assessments, as consistent blinding procedures are typically maintained across laboratory analyses. |
| **Q8** Incomplete outcome data adequately addressed? | **Yes** | Complete datasets are reported for all outcomes, with no indication of missing data or exclusions. |
| **Q9** Free from selective outcome reporting? | **Yes** | All methodological procedures described in the methods section have corresponding results reported, with no apparent omissions. |
| **Q10** Free from other high-risk bias? | **No** | No other significant sources of bias (e.g., ethical issues, confounding funding, design flaws) are reported, and the study follows standard experimental and reporting practices. |

**Wang et al. 2025** (5)

| Q | Binary response | Justification |
|---|---|---|
| **Q1** Allocation sequence adequately generated/applied? | **No** | The authors state that mice were "housed in the neurosurgery laboratory" and that the PCL model was prepared, but no description of randomization for group assignment (e.g., control, PCL, PCL+NP) is provided. Allocation sequence generation is not mentioned. |
| **Q2** Groups similar at baseline / confounder control? | **No** | While the study uses age-matched C57/BL6 mice, there is no explicit statement confirming baseline similarity between experimental groups (e.g., weight, health status) or adjustment for potential confounders in the statistical analysis. |
| **Q3** Allocation adequately concealed? | **No** | No mention is made of allocation concealment methods (e.g., sealed envelopes, coded treatments). The absence of such description means concealment cannot be assumed. |
| **Q4** Animals randomly housed? | **Unclear** | The authors state mice were "housed in the neurosurgery laboratory" under standard conditions, but it does not specify whether housing was randomized across treatment groups to avoid cage effects. |
| **Q5** Caregivers/investigators blinded? | **No** | The article does not mention blinding of caregivers or investigators during animal treatment, behavioral testing, or sample collection. Blinding is not described. |
| **Q6** Animals randomly selected for outcome assessment? | **Unclear** | The methods describe how mice were used for behavioral tests and sampling, but no explicit statement confirms that animals were randomly selected from each group for outcome assessment. |
| **Q7** Outcome assessors blinded? | **No** | Blinding of outcome assessors (e.g., during behavioral scoring, histological analysis, or omics data processing) is not mentioned in the methods or results sections. |
| **Q8** Incomplete outcome data adequately addressed? | **Yes** | Results are reported for all described experiments (behavior, histology, proteomics, metabolomics) without mention of missing data or exclusions, suggesting complete outcome reporting. |
| **Q9** Free from selective outcome reporting? | **Yes** | All planned methodologies (behavioral tests, Transmission electron microscopy-TEM, fluorescence imaging, proteomics, metabolomics, HE staining) appear to have corresponding results reported in the figures and text. |
| **Q10** Free from other high-risk bias? | **Yes** | No other significant biases are evident (e.g., ethical approval obtained, no conflicting funding, standard experimental and reporting practices followed). |

**Ma et al. 2024** (6)

| Q | Binary response | Justification |
|---|---|---|
| **Q1** Allocation sequence adequately generated/applied? | **No** | The authors state that mice were obtained and then "mice were gavaged with 1 mg of NPs [...] every day for 28 days, and the control mice were provided with the equal volume of $ddH_2O$". No method of randomization (e.g., random assignment to groups) is described. |
| **Q2** Groups similar at baseline / confounder control? | **Yes** | The study used male BALB/c mice of the same age (4 weeks) and noted that there were no significant differences in body weight, brain index, food, or water intake between groups at baseline, indicating baseline similarity. |
| **Q3** Allocation adequately concealed? | **No** | No mention is made of allocation concealment procedures (e.g., sealed envelopes, coded treatments). The absence of such description means concealment cannot be confirmed. |
| **Q4** Animals randomly housed? | **Unclear** | The article states mice were housed under standard conditions but does not specify whether cage assignment was randomized across treatment groups to avoid housing bias. |
| **Q5** Caregivers/investigators blinded? | **No** | The manuscript does not mention blinding of caregivers or investigators during dosing, behavioral testing, or sample collection. |
| **Q6** Animals randomly selected for outcome assessment? | **Unclear** | The authors describe that "mice were subjected to" behavioral tests and tissue collection, but no explicit statement confirms random selection from each group for specific assessments. |
| **Q7** Outcome assessors blinded? | **No** | Blinding of outcome assessors (e.g., during behavioral scoring, histological analysis, flow cytometry, or microscopy) is not mentioned. |
| **Q8** Incomplete outcome data adequately addressed? | **Yes** | Results are reported for all described experiments (behavior, histology, cell viability, mitochondrial assays, etc.) without mention of missing data or exclusions. |
| **Q9** Free from selective outcome reporting? | **Yes** | All planned methodologies (behavioral tests, TEM, fluorescence imaging, cell assays, molecular analyses) appear to have corresponding results reported. |
| **Q10** Free from other high-risk bias? | **Yes** | No other significant biases are evident (e.g., ethical approval obtained, no conflicting funding, standard experimental and reporting practices followed). |

**Teng et al. 2025** (7)

| Q | Binary response | Justification |
|---|---|---|
| **Q1** Allocation sequence adequately generated/applied? | **Unclear** | The methods state that embryos were "kept at a density of 2/mL... following exposure" to different treatment groups, but there is no description of how this allocation was randomized (e.g., computer-generated sequence, random number table). The term "randomly selected" is used for outcome assessment, not for initial group allocation. |
| **Q2** Groups similar at baseline / confounder control? | **Yes** | The study uses a full life-cycle exposure starting with 2-hour post-fertilization (hpf) embryos from the same spawning event. This design ensures all groups originate from the same genetic pool and are developmentally identical at the point of intervention, establishing strong baseline similarity. |
| **Q3** Allocation adequately concealed? | **Unclear** | No procedures are described to conceal the allocation sequence (e.g., sealed envelopes, centralized assignment) from the researchers setting up the exposure tanks. This is a common omission but introduces potential selection bias. |
| **Q4** Animals randomly housed? | **Unclear** | While housing conditions are described, there is no explicit statement that the tanks containing different treatment groups were randomly positioned within the flow-through system to control for environmental gradients (light, temperature, noise) |
| **Q5** Caregivers/investigators blinded? | **No** | There is no mention of blinding during the 120-day exposure period for daily care, feeding, or water changes. The caregivers would necessarily know the treatment groups. |
| **Q6** Animals randomly selected for outcome assessment? | **Yes** | The methods explicitly state that for behavioral tests, "male and female zebrafish were randomly selected from each treatment". This indicates a random sampling process was used for outcome assessment. |
| **Q7** Outcome assessors blinded? | **Yes** | For behavioral experiments, researchers performed analyses using the uEye Cockpit software, which can be considered a method of blinding. |
| **Q8** Incomplete outcome data adequately addressed? | **Yes** | Sample sizes are consistently reported for all endpoints (e.g., n=6 for metabolomics, n=8 for social behavior, n=3 for imaging). There is no report of missing data, exclusions, or dropouts, indicating complete datasets. |
| **Q9** Free from selective outcome reporting? | **Yes** | All methodological endpoints described in Section 2 (behavior, imaging, histology, TEM, qPCR, metabolomics) have corresponding results reported in Section 3 and the figures. No apparent outcomes are missing. |
| **Q10** Free from other high-risk bias? | **Yes** | No other significant biases are evident (e.g., ethical approval obtained, no conflicting funding, standard experimental and reporting practices followed). |

**Binqi et al. 2024** (8)

| Q | Binary response | Justification |
|---|---|---|
| **Q1** Allocation sequence adequately generated/applied? | **No** | The authors state: "individuals were randomly selected from the stock population for the exposure experiments" (Page 2, Materials and methods). While this indicates a form of random selection, it does not describe a specific, robust method for generating and applying an allocation sequence (e.g., randomization table, software) for assigning fish to specific treatment groups (control, 25 µg/L, 250 µg/L). The description is insufficient to confirm adequate sequence generation. |
| **Q2** Groups similar at baseline / confounder control? | **Yes** | The groups were derived from the same stock population and randomly selected, implying baseline similarity. Furthermore, the results state: "No obvious mortality or significant changes in weight or length were observed in zebrafish exposed to PS-MPs over the 40 days ($p > 0.05$, Fig. S2)" (Page 3, Results, line ~107). This confirms no significant baseline differences in key physical parameters. The analysis did not require adjustment for confounders as groups were comparable |
| **Q3** Allocation adequately concealed? | **No** | The manuscript does not mention any procedures for concealing the allocation sequence from the researchers' assigning zebrafish to treatment tanks. There is no description of methods such as sealed envelopes or central randomization. |
| **Q4** Animals randomly housed? | **Yes** | While not explicitly stated as "randomly housed", the standard protocol following random selection and group assignment implies that zebrafish were housed in their respective treatment tanks (control, 25 µg/L, 250 µg/L). The exposure was tank-based, so housing was intrinsically linked to the treatment group allocation |
| **Q5** Caregivers/investigators blinded? | **No** | The manuscript does not describe any blinding of personnel involved in daily care (caregivers) or during the exposure phase. Investigators preparing and renewing the PS-MPs solutions would necessarily know the treatment groups. |
| **Q6** Animals randomly selected for outcome assessment? | **Yes** | The manuscript states: "Behavioral data were collected from at least 30 individuals per group" (Page 2, Materials and methods). While not explicitly using the word "random", the standard practice for such assays is to test a representative subset from each treatment tank, which implies non-biased selection. For molecular endpoints, sample sizes are given (e.g., "n = 4 per group"), but the method of selecting specific fish for sacrifice is not detailed. |
| **Q7** Outcome assessors blinded? | **Unclear** | For behavioral tests, the analysis was performed using tracking software (Smart v3.0), which can reduce subjective bias. However, the manuscript does not |

| Q | Binary response | Justification |
| --- | --- | --- |
| | | explicitly state whether the person conducting the tests (placing fish in apparatus, recording) or the person analyzing the raw video data was blinded to treatment groups. For histological, transcriptomic, and metabolomic analyses, blinding of assessors is not mentioned. |
| **Q8** Incomplete outcome data adequately addressed? | **Yes** | The manuscript reports specific sample sizes (n numbers) for all experiments (e.g., n=30 for behavior, n=4 for qPCR, n=6 for ELISA). There is no mention of animals being excluded from analysis or data being missing. The results appear to be based on complete datasets. |
| **Q9** Free from selective outcome reporting? | **Yes** | The study reports a comprehensive set of outcomes aligned with its aims: behavioral tests (NTT, LDB, ST), histology, transcriptomics (RNA-seq and qPCR validation), metabolomics, and in vitro cell assays. Both positive findings (e.g., behavioral changes, gene upregulation) and negative/non-significant results (e.g., no change in tnf-a, no effect on cell viability) are reported. |
| **Q10** Free from other high-risk bias? | **Yes** | No other significant biases are evident (e.g., ethical approval obtained, no conflicting funding, standard experimental and reporting practices followed). |

**Feng et al. 2024** (9)

| Q | Binary response | Justification |
|---|---|---|
| **Q1** Allocation sequence adequately generated/applied? | **No** | The authors do not describe how fish were allocated to experimental groups. No randomization method or sequence generation process is mentioned. |
| **Q2** Groups similar at baseline / confounder control? | **Yes** | The authors state that all fish were four-month-old marine medaka reared under identical conditions before exposure, implying baseline similarity. Statistical analyses (ANOVA) were used to compare groups without explicit adjustment for confounders, but the experimental design suggests comparable starting conditions. |
| **Q3** Allocation adequately concealed? | **No** | There is no mention of allocation concealment procedures in the methods section. |
| **Q4** Animals randomly housed? | **No** | The authors do not state that housing was randomized. They mention replicate tanks per group but not random assignment of tanks or animals to housing conditions. |
| **Q5** Caregivers/investigators blinded? | **No** | Blinding of caregivers or investigators is not mentioned in the methods. |
| **Q6** Animals randomly selected for outcome assessment? | **Yes** | The methods state that five adult fish or twenty larvae from each group were used for behavioral tests, but it is not explicitly stated that they were randomly selected. However, standard practice in such studies often implies random selection, though not explicitly confirmed. |
| **Q7** Outcome assessors blinded? | **No** | No information is provided regarding blinding of outcome assessors during data collection or analysis. |
| **Q8** Incomplete outcome data adequately addressed? | **Yes** | The study reports data from multiple generations and stages, with no indication of missing data or dropouts. All measurements described appear complete. |
| **Q9** Free from selective outcome reporting? | **Yes** | The authors present results across multiple endpoints (behavior, oxidative stress, metabolism, development) as outlined in the objectives, with no obvious omission of planned outcomes. |
| **Q10** Free from other high-risk bias? | **Yes** | No other significant biases are evident (e.g., ethical approval obtained, no conflicting funding, standard experimental and reporting practices followed). |

**Luo & Lin 2025** (10)

| JBI Item | Judgment | Justification |
|---|---|---|
| Inclusion criteria clearly defined | Low risk | Eligibility criteria explicitly stated (≥18 years, on-campus students). |
| Study subjects and the setting described in detail | Low risk | Demographic and clinical characteristics adequately reported. |
| Exposure measured in a valid and reliable way | **High risk** | Microplastics exposure estimated using literature-based concentration values rather than individual-level measurements. |
| Standard criteria used for outcome measurement | Low risk | Depressive symptoms assessed using validated Chinese version of PHQ-2. |
| Confounding factors identified | Low risk | Major demographic and lifestyle confounders identified. |
| Strategies to deal with confounding | Moderate risk | Multivariable logistic regression applied; however, residual confounding (e.g., socioeconomic status, academic stress, prior psychiatric history) cannot be excluded. |
| Outcomes measured in a valid and reliable way | Low risk | Standardized self-administered questionnaire with established psychometric properties. |
| Appropriate statistical analysis | Low risk | Logistic regression models, quartile analysis, restricted cubic splines appropriately applied. |

**References**


1. Su Z, Kong R, Huang C, Wang K, Liu C, Gu X, et al. Exposure to polystyrene nanoplastics causes anxiety and depressive-like behavior and down-regulates EAAT2 expression in mice. Arch Toxicol. 2025 Jun 1;99(6):2595–609. doi:10.1007/s00204-025-04002-6

2. Shin HS, Lee SH, Moon HJ, So YH, Lee HR, Lee EH, et al. Exposure to polystyrene particles causes anxiety-, depression-like behavior and abnormal social behavior in mice. Journal of Hazardous Materials. 2023 Jul 15;454:131465. doi:10.1016/j.jhazmat.2023.131465

3. Liu Q, Hu W, Zhang Y, Ning J, Pang Y, Hu H, et al. Comprehensive Analysis of lncRNA-mRNA Expression Profiles in Depression-like Responses of Mice Related to Polystyrene Nanoparticle Exposure. Toxics. 2023 Jul 10;11(7):600. doi:10.3390/toxics11070600 PubMed PMID: 37505566; PubMed Central PMCID: PMC10386552.

4. Guo Y, Li M, Liu X, Duo H, Huang B, Lu H, et al. Perinatal exposure to polystyrene nanoplastics alters socioemotional behaviors via the microbiota-gut-brain axis in adult offspring mice. Brain Behav Immun. 2025 Aug;128:121–33. doi:10.1016/j.bbi.2025.04.002 PubMed PMID: 40187670.

5. Wang L, Ma JQ, Song LJ, Qu XP, Zhang Y, Fan HM, et al. Comprehensive multi-omics, behavioral and morphological analysis of the hazards of nano-plastics in mice with internal carotid artery occlusion. Ecotoxicology and Environmental Safety. 2025 Jan 1;289:117711. doi:10.1016/j.ecoenv.2025.117711

6. Ma Y, Xu D, Wan Z, Wei Z, Chen Z, Wang Y, et al. Exposure to different surface-modified polystyrene nanoparticles caused anxiety, depression, and social deficit in mice via damaging mitochondria in neurons. Sci Total Environ. 2024 Apr 1;919:170739. doi:10.1016/j.scitotenv.2024.170739 PubMed PMID: 38340854.

7. Teng M, Li Y, Zhao L, White JC, Sun J, Zhang Z, et al. Life cycle exposure to differentially charged polystyrene nanoplastics leads to gender-specific particle accumulation and neurotoxicity in zebrafish (Danio rerio). Environ Int. 2025 Apr;198:109441. doi:10.1016/j.envint.2025.109441 PubMed PMID: 40209392.

8. Yang B, Han Y, Hu S, Xie X, Zhu X, Yuan L. Polystyrene microplastics induce depression-like behavior in zebrafish via neuroinflammation and circadian rhythm disruption. Sci Total Environ. 2025 Jan 10;959:178085. doi:10.1016/j.scitotenv.2024.178085 PubMed PMID: 39708463.

9. Feng JX, Liu B, Chen CZ, Ma YQ, Wang CL, Xu YN, et al. Multigenerational effects of combined exposure of triphenyltin and micro/nanoplastics on marine medaka (Oryzias melastigma): From molecular levels to behavioral response. J Hazard Mater. 2024 Dec 5;480:136365. doi:10.1016/j.jhazmat.2024.136365 PubMed PMID: 39488982.

10. Luo J, Lin S. Association between microplastics exposure and depressive symptoms in college students. Ecotoxicology and Environmental Safety. 2025 Apr 15;295:118142. doi:10.1016/j.ecoenv.2025.118142
